# Growing Pains at Hospitals: Opportunities and Issues of Service Expansion in Maximum Care

**DOI:** 10.3389/fmed.2017.00090

**Published:** 2017-06-28

**Authors:** Juergen Hinkelmann, Joachim Paul Hasebrook, Thomas Volkert, Klaus Hahnenkamp

**Affiliations:** ^1^University Hospital Frankfurt, Frankfurt, Germany; ^2^zeb.business school at Steinbeis University Berlin, Berlin, Germany; ^3^Department of Anesthesiology, Intensive Care Medicine and Pain Management, University Hospital Muenster, Muenster, Germany; ^4^Department of Anesthesiology, Intensive Care Medicine, Pain Management and Emergency Medicine, University Hospital Greifswald, Greifswald, Germany

**Keywords:** anesthesiology, human resources, skill mix, staff capacity, staff fluctuation

## Abstract

**Purpose:**

Due to the demographic change morbidity raises the demand for medical hospital services as well as a need for medical specialization, while economic and human resources are diminishing. Unlike other industries hospitals do not have sufficient data and adequate models to relate growing demands and increasing performance to growth in staff capacity and to increase in staff competences.

**Method:**

Based on huge medical data sample covering the years from 2010 to 2014 with more than 150,000 operations of the Department for Anesthesiology at the University Hospital Muenster, Germany, comparisons are drawn between the development of medical services and the development of personnel capacity and expertise.

**Results:**

The numbers of surgical operations increased by 21% and “skin incision to closure” time by 17%. Simultaneously, personnel capacity grew by 16% largely resting upon recruiting first-time employees. Expertise measured as “years of professional experience” dwindled from 10 years to 5.4 years on average and staff turnover accelerated.

**Conclusion:**

Static benchmark data collected at fixed reference dates do not sufficiently reflect the nexus between capacity and competence and do not reflect the dynamic changes in a hospital’s requirements for expertise and specialization, at all. Staff turnover leads to a loss of experience, which jeopardizes patient safety and hampers medical specialization. In consequence of the dramatic shortage of medical specialists, drop-off rates must be reduced and retention rates must be increased. To that end, working conditions need to be fundamentally converted for a multigeneration, multicultural, and increasingly female workforce.

## Introduction

Demographic development will bring about a constantly growing need for medical treatment of long-term and chronically ill patients as well as geriatric medicine. At the same time, the proportion of the economically active population will shrink, thereby making it more difficult to fund the health system. For example, demand forecasts in Germany for case figures and stay length until 2030 indicate an increase in case figures in hospitals from 19.4 million to 22 million cases of treatment while the group of patients over 60 years will increase from 51 to 61% (1–3). The amount of acute hospital beds has dropped in most OECD countries from 4.7 beds per 1,000 inhabitants in 1995 to 3.8 beds in 2007 (4). The reduction of hospital beds in many countries entails fewer cases of hospitalization and shorter average stays (5). In almost all OECD member states, costs in the health-care sector represent the largest expenses (6, 7). The productivity pressure is thus higher depending on how specialized the medical treatment is and the associated expense increases: in Germany, during the last few years more patients being treated in less time and with fewer facilities (8). In many innovation-dependent industries, the relationship between full-time equivalents (FTEs), employee expertise, and framework conditions for corporate development has been discussed in terms of three main approaches (9–11):

1. Strategic positioning of the company in order to attract and retain talent (12) as well as a learning orientation in order to build an efficient workforce (13).
2. Systematic human resource and performance management in order to ensure a competitive edge (14) as well as the growth of intellectual and corporate capital (15).
3. Knowledge and competence management as the central task of a company in order to ensure the capacity to remain competitive and adaptive (16).

Especially regarding the last two perspectives medical research has not yet combined economic growth and medical performance in dependency to the requirements of personnel capacity and competence in University Hospitals. This research is needed in order to examine economic efficiency in the provision of medical services (17). Because health systems are facing greater scarcity of financial and human resources, high quality medical treatment can only be ensured by effectively utilizing resources (18). Productivity certainly is the predominant parameter for measuring efficiency and quality of medical treatment (19).

### Falling Physician Capacities and Expertise

In almost all OECD countries, the physician densities increased by an average of 2% annually between 1990 and 2007 (20). The Association of American Medical Colleges predicts that the United States will need 26% more physicians over the period 2006–2025. However, the calculated supply of physicians will increase by only 10–12% (21, 22). Due to demographic change and the increase in non-transmitted diseases, OECD countries should expect a 10–15% increase in morbidity by 2030. The OECD member states, therefore, see a growing demand for specialist personnel in the health-care sector (23).

### Special Circumstances at German University Hospitals

University hospitals are at the forefront of maximum medical care and take on patients who have difficult or complicated illnesses. In addition, university hospitals are also engaged in research, student training, and further training for specialist physicians (24). Further medical training is associated with additional personnel costs, because junior staff may not be placed in all positions and the positions that they fulfill are not fully productive. Expenses for supervision and instruction through the chief residents and specialists are on top.

### Research Issues

#### Comparable Measures of Service Expansion

The Department of Anesthesiology, Intensive Care and Pain Medicine at the University Hospital Muenster (UKM), University of Muenster has recorded comprehensive performance and capacity data for years. Based on these data and additional surveys, the relationship between expanded services and increasing capacity and expertise needs will be tested.

#### Benchmark Data for Personnel Capacity and Performance Indicators

General-purpose data (25) provided by international databases, such as Eurostat, WHO, and OECD, cannot be used as reliable trend analyses, performance and outcome comparisons for OP management in hospitals (26). Even at the 33 university hospitals in Germany there has been no success despite many years of trying to reach a standard.

#### Comparison of Capacities and Expertise

As standardized performance indicators have not yet been defined, there is no single standard but a great variety of models for medical workforce planning in use (27–29). Most models deduce capacity and skill mix either from performance or from revenue indicators (30). However, average duration of services and anesthesia bonding times are hard to predict and cost rates for diagnosis related group (DRG) are mixed calculations without the details needed for proper workforce planning (31).

#### Gap between Increase in Services and Increase in Capacities and Expertise

Increases to medical productivity are not just in terms of capacities but also the level of expertise (32). Due to legal and professional regulations direct assessments may not always be enforced (33). Therefore, indirect measures, such as years of work experience, level of professional training, and additional qualifications, are employed (34).

The data analyzed in this article should help in finding comparable measures of service expansion, identify possible benchmark data for increase in medical performance and staff capacity, and examine possible gaps between increase in services and increase in capacities and levels of expertise, which might jeopardize efficiency and quality in medical care.

## Materials and Methods

### Measuring Service Development

As a data basis, we have used the last 5 years of development of the operational services at the UKM. As a benchmark for this performance increase, we selected the time from skin incision to closure both during (RWH) and outside of the regular working hours (NRWH). The time from skin incision to closure describes the duration between making an incision into the skin and final suture stitch made on the patient—it thus represents the pure operation time. Besides the time from skin incision to closure, we use the amount of operations conducted in order to better evaluate the service growth: if the skin-incision-to-closure-time increases, but the number of operations does not, then this indicates more complex and thus more time-consuming operations; if the skin-incision-to-closure-time and the number of operations increase, then there is an increase due to case number growth. It is not clear whether the complexity of operations has changed. We compare the service figures of development of economic case severity through the case mix index (CMI), the change of service provision in anesthesiology through the number of anesthesia cases and the minutes under anesthetics and the average duration of anesthesia for the years 2010–2014. Minutes under anesthetics includes the time from the beginning of the anesthesia upon the arrival of the patient until the end of the anesthesia upon the departure of the patient, e.g., to the recovery room.

### Comparing Service and Capacity Growth

To compare with the service growth, we use the personnel development at the Department of Anesthesiology, Intensive Care and Pain Medicine at the UKM in the same period. The development of the amount of anesthesiologists is monitored by means of FTEs. FTE represents the amount of work that a full-time worker completes within a comparable period at a workload of 100%. If a group of staff is made up of personnel with different levels of occupation (including part-time staff), the workload is proportionally adjusted by time to give the equivalent workload of full-time employees. Because there are personnel changes at the start and end of the month due to leavers and new staff, the records for personnel levels are made on the 15th of each month. The development of FTEs for residents, specialists, and attending specialist will be monitored while considering downtimes due to on-call duty and sick leave.

### Comparing Personnel Capacities and Expertise

We identify the evolution of personnel expertise by comparing the amount of hospital’s new staff and leavers broken down into residents, specialists, and chief residents for the same 5-year period. We compare the changes of the various medical groups as an index. Besides the group that the physicians belong to, medical expertise is particularly linked to occupational experience (35). We thus also record the “licensing age,” i.e., the amount of time since the license to practice medicine—which is also the requirement for training as a specialist—was obtained. As is the case for group belonging, the “licensing age” as a measure of occupational experience is only an indirect indicator for acquired expertise.

## Results

### Operational vs. Anesthesiological Service Development

**Figure 1:**
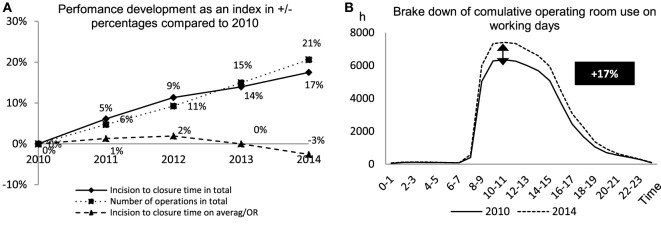
**(A)** Incision to closure time, number of operations, and incision to closure time per operating room as benchmark for performance development compared to 2010. **(B)** Cumulative use of the operating rooms measured in hours in relation to working hours of a day to benchmark when the performance increase took place.

The operational services have been increased continuously at the UKM since 2010: while the cumulative time from skin incision to closure was 2,260,672 min in 2010, this has been expanded dramatically in recent years. In 2014, skin-incision-to-closure-time was 2,656,212 min, which represents an increase of 17%. The increase of skin-incision-to-closure-time is not only generated by an extension of surgery times but also through the increased number of operations conducted (see Figure 1A): the number of operations at UKM was 31,674 in 2010, while this reached 38,203 in 2014 (increase of 21%). The average skin-incision-to-closure-time per operation remained rather constant during the reporting period (decrease of 3%). This service development was due to consolidation within regular working hours (RWH). In 2009, the RWH in anesthesiology were adjusted according to times of operating room use. Figure 1B illustrates the distribution of the cumulative operating room use for the years 2010 and 2014 broken down by times. The greatest increase of performance was in the operating room use during RWH between 8:00 a.m. and 3:00 p.m. Outside of the RWH, between 3:00 p.m. and 7:30 p.m., the increased operating room use is less considerable. Use after 7:30 p.m. is almost unchanged.

**Figure 2:**
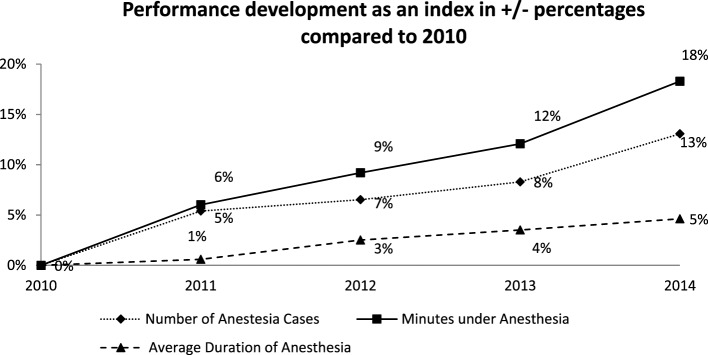
Number of anesthesia cases, minutes under anesthesia, and average duration of anesthesia as benchmark for the performance development compared to 2010.

According to this general service trend, the number of anesthetic cases conducted, the anesthesiology hours per year, and the average length of anesthesia per case all increased (see Figure 2): the number of anesthesia cases increased from 30,257 (2010) to 34,211 (2014). In the same period, the number of anesthesiology minutes provided increased from 4,170,571 to 4,933,620 min in 2014. In doing so, the anesthesia time increased by 18% in the evaluated period, the SNZ increased by 17%. By dividing the anesthesiology minutes per year by the number of cases of anesthesiology, we have found the average anesthetic times per case; this increased from 137.48 min in 2010 to 144.21 min in 2014. This increase does not necessary entail an increase in the complexity of anesthetics. Complex operational interventions also demand an increased level of preparation, e.g., for neuromonitoring and complex surgical positioning, without necessarily changing the complexity of the anesthesiological service. In order to identify a possible increase in complexity of anesthetics, the American Society of Anesthesiologists (ASA) classification from 2010 to 2014 was assessed. The ASA classification sorts patients into various risk groups according to their physical condition. This relationship, however, remains the same upon growing service numbers, which means that there is both an increase in quantitative service numbers and in quality. As an alternative method to measure the complexity of anesthetic cases, we assessed the average initiation time, which hardly changed over the evaluated period.

### Capacity Development

**Figure 3:**
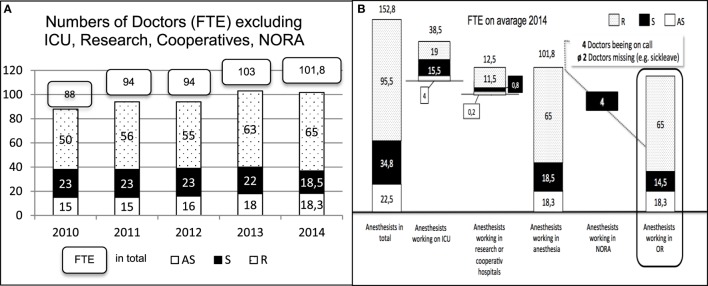
**(A)** Development of the number of anesthetists measured as full-time equivalent (FTE) working only in the operating rooms of the University Hospital Muenster (UKM), Germany. **(B)** Distribution of all Anesthetists working at the Department of Anesthesiology, Intensive Care Medicine an Pain Management, UKM, Germany.

As services increased, personnel was expanded in the UKM’s clinic for anesthesiology, surgical intensive care medicine, and pain management (see Figure 3A): the number of FTEs increase from 88 in 2010 to 101.8 in 2014 (increase of 16%). The figure only considers employees for clinical anesthetics in the surgery areas. The figure does not include personnel for intensive-care medicine, external locations such as hospitals in the training association or non-operating room anesthesia (NORA). The capacity growth is particularly related to the employment of residents (increase of 30%). With a significantly lower number, the growth of chief residents matches the total growth (increase of 22%). Because chief resident positions are mostly staffed with specialists from within the institute and open chief resident positions could not be staffed through the “next generation” of training assistants or external applicants, the number of specialists decreased by 20%.

If you look at the number of 152.8 anesthetists worked the time of assessment in the Department of for anesthesiology, surgical intensive care medicine, and pain management at the UKM, 95.5 were residents, 34.8 were specialists, and 22.5 were attending specialists. After staffing the operational intensive care departments, NORA, positions in the training association, research and teaching, there remain 101.8 physicians for clinical anesthesia (18.3 attending specialists, 18.5 specialists, and 65 residents). In clinical anesthesiology, four non-operational workstations outside of the surgery departments need to be staffed by specialists each day, because supervision by an attending specialist or a specialist nearby is not possible at these stations. In addition, four specialists are needed each day for staffing the on-call duties.

In 2014, the average downtime due to sickness of physicians in anesthesiology was ~3.6%, meaning that on average two anesthesiologists were sick per day. As a result, from the 34.8 specialists in the department, there were effectively 14.5 available for surgical services. In the day-to-day work in a hospital with 37 operating rooms that are partially occupied in a two-shift system and in which approximately 130 cases of anesthetics are conducted each day, there are, however, only 9–10 specialists available each day (see Figure 3B).

### Expertise Development

**Figure 4:**
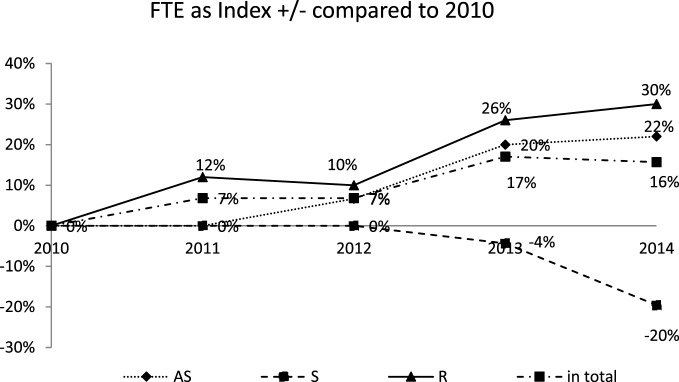
Development of the amount of full-time equivalent (FTE) compared to 2010 distinguished between attending specialist (AS), specialist (S), and resident (R) as well as the change in total.

As indicated above, the amount and complexity of anesthesiological services and thus also the requirements for medical expertise have increased during the evaluated period. For this reason, the evaluation of the capacity development needs to be expanded to include an assessment of expertise development. The work groups of attending specialist, specialists, and residents, whose development has already been indicated (see Figure 4), are also directly linked to the levels of expertise. Attending specialists are responsible for supervising and training the residents. Because the number of residents has increased much quicker than the number of attending specialists, they are now responsible for almost four (3.6) residents rather than slightly more than three (3.1).

During the evaluated period, the number of new recruits increased from 13 anesthetists in 2009 to 31. Although the number of leavers also increased at the same rate, the balance of recruits and leavers remained positive in each year. Because the number of residents has grown faster than the other groups of medical staff, their structure has shifted to lower levels of expertise. Another important indicator of expertise is how many years of relevant work experience exist. We have thus adjusted the fluctuation balance into “years of experience,” that is, gains and losses of years of medical work through new recruits or leavers. In 2009, “years of experience” were gained through 13 new recruits and 6 leavers, while in 2014, despite significantly more new recruits (42), “years of experience” were lost.

**Figure 5:**
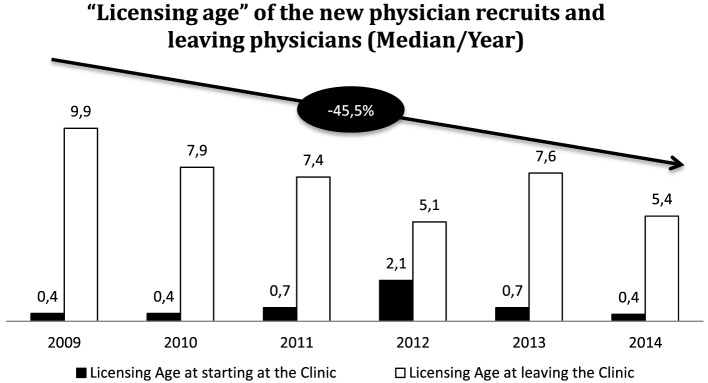
Licensing age at starting and at leaving the clinic as indicator for work experience compared from 2009 to 2014. It compares how long a doctor stays after he graduates as a specialist (usually after 5 years) and with how much work experience the leavers are compensated by new appointments.

We evaluated the speed of the gains or losses of years of medical work and experience by means of the “licensing age.” Licensing refers to the license to practice medicine awarded upon completion of the theoretical and practical training to become a physician. In order to become a specialist in a particular field, a further 5–6 years of professional training are added. Figure 5 shows that in 2009, specialists continued to work in the hospital for almost 10 years after licensing. In 2014, this was only 5.4 years, i.e., only a short time after completing further training to become a specialist. This led to a 45.5% increase in the loss of years of medical work. This loss is not compensated through increasing occupational experience of new recruits, which varied between one half and two years during the evaluated period.

## Conclusion

### Overview of Results

#### Measures of Service Expansion

During the evaluation period of 2010–2014, the operational services at UKM were increased by 17% mainly by expanding the regular working hours followed by anesthesiological and operational performance increases, with both 18% more anesthesiology minutes and 18% more cases of anesthetics. Qualitative service expansion could not be ascertained: the ASA classifications, which might indicate a change in case complexity, have not significantly changed as well as the CMI, which is often wrongly used for measuring complexity (36, 37). CMI is the total of the relative value for each patient case charged by a hospital in a certain period (effective case mix) divided by the number of cases. The CMI is thus an expression of economic case severity and makes it possible to compare the cost structures of hospitals. However, it hardly reflects whether anesthetics have become more complex and require a higher level of specialization. An increase in complexity is reinforced by a combination of different facts: although the time from skin incision to closure remained constant, the average anesthesiology minutes per conducted surgery increased. At the same time, the average induction time for anesthesia as well as the number and level of complications and complaints stayed the same. Combining these facts we consider the increased duration of anesthesia to be an indicator for the increasing complexity.

#### Benchmarking Capacity, Performance, and Expertise

Although the capacities of medical employee groups increased by 16%, this figure mainly concerns residents who cannot and may not take over the responsibilities of specialists, and who also need to be supervised and trained by chief residents. This represents a major additional burden on top of medical treatment, but is indispensable for developing expertise. The ratio of chief residents to residents has worsened from 3.1 to 3.6, which can have a negative influence on the quality of the medical work and training (38). The German Association for Anesthesia and Intensive Care (DGAI) and the Association of German Anesthesiologists (BDA) recommend at least one chief resident in order to supervise up to seven residents. In areas of higher complexity or perioperative risk, e.g., cardio anesthesia, the ratio needs to be cut down to one experienced specialist to two or three residents.

#### Increase in Services and Increase in Capacities and Expertise

We argue that University Hospitals performing maximum medical care with an increasing specialization and number of emergency should restrain the ratio of specialist to residents to a minimum (39). Out data have shown that physicians are leaving the hospital earlier once achieving the specialist qualification, while the “licensing age” of new recruits cannot be significantly increased to compensate for the loss of years of experience: expertise is being lost while the personnel capacity is increasing.

## Discussion of Results

In Germany, the number of physicians and the expansion of services have grown both at approximately 20%. As the average age of hospital physicians increased, the number of years of employment until age-related retirement dropped (40). In consequence, the average age of both panel physicians and ward physicians continually increased (41). This “loss of remaining professional years” is also a loss of “years of experience.” Taking the increasing amount and complexity of cases as a basis, there will be a shortage of 47,000 full-time physicians working in hospitals by the year 2030 (42). The demand cannot be covered by hidden reserves on the labor market because there is full employment for jobs in German human medicine and interdisciplinary compensations are hardly possible (43). In addition, legal requirements being specified by the final instance, the German Federal Court of Justice (Bundesgerichtshof), add to the shortage of anesthetists: anesthetics may only be issued by a specialist who has been certified as an anesthesiologist, or at least under the immediate supervision with both visual and verbal contact of another physician (44, 45). In all cases, patient safety must have the highest priority (46). Because supervision cannot be ensured at anesthesia workstations outside of the operating room (NORA), all NORA workplaces have to be filled with specialists, who are then not available in the operating rooms. Under these circumstances, optimization potentials are low and only an increase in capacities and expertise of the medical staff ensures unproblematic performance increases (47, 48). Not only medical services contribute to the work load but also professional development and training. Training costs are not subsidized in the German DRG system, even though they represent a significant extra burden (49). Cutting training costs is not an option, because it is not only needed to ensure further specialization in medical services and guarantee patient safety, but it is also the most import factor of employee retention (50, 51).

Medical management takes into performance indicators, skill mix, and quota for absents (33).

But static benchmarks to key dates do not sufficiently take into account the hospital reality as dynamic changes. Besides an increase in capacity, the level of expertise must also be enhanced: not merely can losses of experience be compensated through the recruitment of young and inexperienced medical staff, but rather these recruits add to the burden of experienced medical staff in terms of training and supervision. In addition, the young medical staff is predominantly female: female physicians will clearly outnumber their male colleagues in nearly all European countries in the near future (23). Even the share of female medical students in the majority of the European countries will overtake that of males in upcoming years (52). In Germany, already 70% of medical students are female^1^. It still is unclear what the special demands of female medical doctors and what are the implications on creating an accepted and more employee-orientated working environment in hospitals. The poor adaptation of working conditions in hospitals to demands and expectation of medical staff as already led to an increased level of part-time work (53), work-related stress (54), dissatisfaction with working hours, career perspectives and remuneration (55), and—finally—escape from curative activities (56). In summary, the gap between demand for top medical services and readily available capacities and competence of physicians is constantly widening.

## Recommendations and Further Research

Qualitative and quantitative performance increases need to be monitored by means of suitable indicators, while the appropriate personnel needs—in terms of capacity, levels of expertise and occupational experience—need to be considered. Demographic change will generally make this more important as it entails the higher needs to take action, fewer junior physicians, and thus a greater workload. Once this vicious circle of continuous service expansion, higher workload, and higher willingness to change jobs have established, no amount of new recruits will be able to ensure the same quality of patient care; instead, improved retention of specialists and other highly qualified medical staff will be necessary.

The German Federal Ministry of Education and Research (BMBF) promotes the “FacharztPlus” initiative, which tackles this issue (57). In order to create attractive working conditions for staff in hospitals, a noticeable improvement and further training are needed. The “FacharztPlus” initiative is evaluating ways to retain physicians at the workplace after they are awarded their specialist qualification. To do so, the appropriateness of further qualifications to be acquired alongside the professional career and an expertise-related work organization based on different stages of life are determined, while a more flexible, tailored work organization is evaluated for life-long perspectives. Long-term perspectives after professional training are not just attractive for retention but also for recruiting specialists. Based on the project results, training and IT planning offers that are tailored to clinics are desired. The project compares the management of medical services in the hospital with the service management of other sectors in order to develop effective and practicable solutions. The “FacharztPlus” initiative will thus develop an implementation plan for combining the conditions for work, utilization, and learning at university hospitals and other hospitals. The results will contribute to adapting working conditions to the demographic change, the resulting development of services and the multifaceted changes for people involved in curative activities. Better employee retention provides for higher availability of expertise needed to perform maximum medical care in university hospitals. Further research is needed to develop and test measures to substantially reduce staff turnover and improve retention among physicians working in hospitals.

In order to ensure high-quality medical care in the medium and long term, demands of the young generation of physicians must be regarded: the number of female physicians in university hospitals will increase, as their working conditions as physicians and researchers will improve—thus, turning a “vicious” into a “virtuous cycle.” We believe that “feminization in medicine” is chance and not a thread, from which both will benefit, men and women. Therefore, further research must address gender issues as a vital part of hospitals’ working conditions. The Medical Women International Association put it like this: *“Society must move beyond blaming women physicians for the unpleasant changes that have affected the practice of medicine, but rather acknowledge the true reasons, which are lack of resources and third-party control. Bright minds are not going to be attracted to a profession that fails to provide job satisfaction, status, influence and lack of monetary reward”* (58).

## Author Contributions

JH is a consultant to the executive board of the University Hospital Frankfurt, Germany. JPH is a professor for human capital management and research director of zeb.business school at Steinbeis University Berlin. TV is attending anesthesiologist and responsible for the department’s medical documentation and economic aspects of anesthesia and intensive care at the Department of Anesthesiology, Intensive Care Medicine and Pain Management at the University Hospital Muenster, Germany. KH is a professor of anesthesiology and director of the department of Anesthesiology, Intensive Care Medicine, Pain Management and Emergency Medicine at the University Hospital Greifswald, Germany.

## Conflict of Interest Statement

The authors declare that the research was conducted in the absence of any commercial or financial relationships that could be construed as a potential conflict of interest.

## Footnotes

^1^https://www.destatis.de/DE/ZahlenFakten/Indikatoren/LangeReihen/Bildung/lrbil05.html?cms_gtp=152382_list%253D1&https=1.
